# Precision medicine for patients with gastro-oesophageal cancer: A subset analysis of the ProfiLER program

**DOI:** 10.1016/j.tranon.2021.101266

**Published:** 2021-11-15

**Authors:** Philippe A. Cassier, Clémentine Peyramaure, Valery Attignon, Lauriane Eberst, Camille Pacaud, Sandrine Boyault, Françoise Desseigne, Mathieu Sarabi, Pierre Guibert, Pauline Rochefort, Nathalie Marques, Michel Rivoire, Aurélien Dupré, Patrice Peyrat, Catherine Terret, Isabelle Ray-Coquard, Clélia Coutzac, David Pérol, Jean-Yves Blay, Olivier Trédan, Christelle de la Fouchardière

**Affiliations:** a Département de Cancérologie Médicale, Centre Léon Bérard, 28 rue Laennec, Lyon 69008, France; b Service d'Oncologie, Centre Hospitalier Universitaire de Limoges, Limoges, France; c Département de la Recherche Translationelle et de l'Innovation, Centre Léon Bérard, Lyon, France; d Institut de Cancérologie de Strasbourg, Strasbourg, France; e Service d'Onco-Hémato Pédiatrie, Hôpital Hautepierre, Centre Hospitalier Universitaire de Strasbourg, Strasbourg, France; f Département de Chirurgie, Centre Léon Bérard, Lyon, France; g Université Claude Bernard Lyon I, Lyon, France; h Direction de la Recherche Clinique et de l'Innovation, Centre Léon Bérard, Lyon, France; i Unicancer, Paris, France

**Keywords:** Oesophageal cancer, Gastric cancer, Molecular alterations, molecular-targeted agents, NGS, CGH

## Abstract

**Background:**

Chemotherapy, anti-HER2 and PD-1 antibodies are standard treatments but only a minority of patients derive long-term benefit from these agents.

**Methods:**

In this report we describe the mutational landscape and outcome of patients with gastroesophageal cancers enroled in the ProfiLER program.

**Results:**

Adenocarcinoma (*n* = 86, 59%), signet-cell (*n* = 37, 25%) and squamous-cell (*n* = 21, 14%) were the dominant histology amongst 147 patients. Genomic analyses could be performed for 114 (78%) patients. The most common genomic alterations involved ERBB2 (15%), KRAS (12%), CCND1 (7%), FGFR1–3 (8%), EGFR (5%) and MET (3%), TP53 (51%) and CDKN2A/B (10%). ERBB2, MET and FGFR alterations were found exclusively in the adenocarcinoma and signet-cell subtypes, while CCND1 amplification, TP53 mutations and CDKN2A/B loss were found in both adenocarcinoma and squamous-cell subtypes. Nine patients (8%) received therapy matched to their genomic alteration, with 5 of them achieving disease control. In an exploratory analysis, patients with stage IV disease at diagnosis who had an actionable alteration had longer overall survival compared to those without.

**Conclusion:**

Genomic profiling for patients with advanced gastroesophageal cancers allows the identification of actionable alterations in large proportion of patients. Increased accessibility to molecularly matched therapy may improve survival in this disease.

## Highlights

• Cancers originating in the stomach and oesophagus have poor prognosis and limited treatment options.
• Gastroesophageal cancer frequently harbour actionable genomic alterations.
• Our data suggest that patients with advanced gastroesophageal cancers and actionable alterations have prolonged survival compared to those who do not.
• Comprehensive genotyping, beyond determination of the HER2/ERBB2 status should be implemented early in the management of patients with gastroesophageal cancers.

**Novelty & impact statement**

Cancers originating in the stomach and oesophagus have poor prognosis and limited treatment options. Gastroesophageal cancer frequently harbour actionable genomic alterations. Our data suggest that patients with advanced gastroesophageal cancers and actionable alterations have prolonged survival compared to those who do not. Comprehensive genotyping, beyond determination of the HER2/ERBB2 status should be implemented early in the management of patients with gastroesophageal cancers.

Alt-text: Unlabelled box

## Introduction

Oesophageal and gastric cancer are common malignancies of the upper gastrointestinal tract with more than 1.5 million new cases estimated in 2018 worldwide. Both are associated with a high disease-related mortality, resulting in similarly high rate of annual deaths (1.3 million). Because of their anatomical proximity, both tumour types share some risk factors and epidemiological features, but also display distinct geographical and temporal patterns in incidence [1].

Oesophageal cancer can be subdivided into squamous cell carcinomas, which predominate in the upper and middle third of the oesophagus and adenocarcinoma which make up the majority of cases in the lower third of the oesophagus. Gastric cancer can also be divided into two distinct subgroups based on anatomical location: gastroesophageal junction (GEJ) and gastric cancer which are dominated by the adenocarcinoma histology, but differ in aetiologies and molecular characteristics [9]. Large scale sequencing efforts have identified several potentially actionable targets in gastric cancer [3,4], but so far, only trastuzumab, which targets HER2 has been widely approved and used in gastro-oesophageal cancer (GOC) overexpressing HER2. Other emerging targets for GOC include microsatellite instability, MET [8] and alterations of FGFR1–3. Despite these recent improvements in molecular classification, cytotoxic chemotherapy remains the backbone of systemic therapy in both the localised and advanced setting, and the responses are in most cases short lived with second and further lines of therapy options still limited.

Prospective use of sequencing to identify actionable target is an ongoing effort of the oncology community but has so far led to only modest results: despite actionable alteration being found in approximately 40% of patients, 20% of patients actually receive matched therapy and only about 10% of these have an objective response (2% of the overall population) [6,13,22,23], though some authors have reported much higher rates of success [19]. In addition, reports from The Cancer Genome Atlas and the International Cancer Genome Consortium have shown over the last decade that the distribution of molecular alterations varies significantly amongst diseases and this impacts the frequency of potentially actionable targets across tumour types [20]. Thus, one could expect that the utility of clinical sequencing could vary between tumour types. Here, we report the outcome of patients with gastro-oesophageal carcinoma who were prospectively enroled in the ProfiLER 01 program.

## Patients and methods

### Study design and procedures

The ProfiLER01 program is a multicentric, prospective and non-randomised on-going study dedicated to patients with advanced/metastatic cancer who progressed after at least one line of standard treatment. Detailed methodology for this study has been previously described [22]. Briefly, after patients provided written informed consent, tumour and blood samples, as well as clinical data were collected. Formalin-fixed and paraffin-embedded tumour specimen, from archival samples of primary tumour, relapse, or metastasis, containing ≥30% of tumour cells, or de novo biopsy were used to determine genetic molecular profiles by next-generation sequencing (NGS) using a 69-gene profiler-panel V2 (see Appendix 1), and genome-wide microarray-based comparative genomic hybridization (aCGH) [22]. In subsequent updates of the NGS panel (from September 2017 onwards) substitutions, small indels (Appendix 1) and genome wide copy number variations (CNVs) and losses of heterozygosity were assessed simultaneously using the OneSeq target enrichment (Agilent) and sequenced on a NovaSeq6000 sequencer (Illumina). The minimal DNA input amount needed was 100 ng for NGS and 1.5 μg for aCGH. Some patients had additional molecular analysis including microsatellite analysis by immunohistochemistry (IHC) (evaluation of MMR protein expression including MLH1, MSH2, MSH6 and PMS2) and/or pentaplex PCR-based assays (directed against 5 microsatellite regions with the Promega MSI Analysis System) and targeted RNA sequencing (RNA seq) (with the FusionPlex RNA CTL_V6 kit, Archerdx)(for the purpose of identifying actionable fusions) (Suppl. Data 1)

The ProfiLER01 study was conducted in accordance with Good Clinical Practice guidelines of the International Conference on Harmonization and the Declaration of Helsinki and approved by the Ethics Committee of Lyon Sud-Est IV. All patients provided written informed consent for molecular analyses as well as collection and analysis of clinical data. ProfiLER01 is registered in ClinicalTrials.gov under number NCT01774409. The main entry criteria were: age 18 years or older, any type of solid tumour considered advanced or metastatic, at least one line of therapy for advanced disease, tumour sample (fresh or archival) available. A weekly molecular tumour board gathering medical oncologist, pathologist and molecular biologists reviewed the results of NGS and aCGH in order to identify genomic alterations of interest and recommend treatment with matched molecular-targeted agents (MTA). The molecular tumour board recommended approved MTAs or clinical trial participation with matched therapy.

### Statistical analysis

A total of 3610 patients were enroled in the Profiler program at Centre Léon Bérard between February 2013 (date of study initiation) and February 2020 (data cut-off for this analysis) and could be analysed. The primary end point of the current analysis was to determine the incidence of genomic alterations in patients with oesophageal or gastric cancer. Secondary objectives were to evaluate the impact of genomic alterations on treatment decision, accessibility and efficacy of MTA, as well as on clinical outcome. The analysis on the current sample set were essentially descriptive: qualitative variables were expressed as percentages with confidence intervals when applicable while quantitative variables were expressed as median and range. Comparison of categorical variables were done using the Chi² or student T test where applicable. Overall survival (OS) was calculated from the date of initial diagnosis to the date of death from any cause or date of the last follow-up (censored observation). Progression-free survival (PFS) was measured from the date of treatment initiation (of the relevant line) to the time of disease progression or death (which ever occurred first), or was censored at the last follow-up. Survival distributions were displayed using the Kaplan-Meier method and compared using the Log Rank test.

## Results

### Patients’ characteristics

One hundred and forty seven patients with carcinoma originating in the stomach or oesophagus were identified (of a total of 357 patients with gastric and 248 patients with oesophageal cancer managed at our centre over the same period). Table 1 describes their main characteristics. Briefly, median age at diagnosis was 58 years (range 25–77), the majority of patients were males (104/147, 71%), with good performance status (117/147 patients (80%) were ECOG 0–1)(17). The primary tumour site was evenly distributed between oesophageal, GEJ and gastric cancer (32, 31 and 36%, respectively). The majority of patients had stage IV disease at diagnosis (87/147, 59%). As expected, intestinal type adenocarcinoma, signet cell carcinoma and squamous cell carcinoma were the dominant histological subtypes (59, 25 and 14%, respectively).

**Table 1.** Main clinical characteristics of patients with gastroesophageal cancer enroled in the ProfiLER programs. (*) Other histologies included neuroendocrine carcinoma (*n* = 1) and undifferenciated carcinoma (*n* = 2).

Characteristics	N (147)	%
Gender		
Female	43	29%
Male	104	71%
Age at diagnosis : median (range) in years	58	(25–77)
Age at study entry: median (range) in years	58	(25–82)
Primary tumour site		
oesophagus	47	32%
cardia	46	31%
non-cardia gastric	54	37%
Histology		
Inestinal-type adenocarcinoma	86	59%
Signet-cell carcinoma	37	25%
Squamous-cell carcinoma	21	14%
Other*	3	2%
Stage at diagnosis		
II-III	60	41%
IV	87	59%
Stage at study entry		
Recurrent	4	3%
Metastatic	143	97%
Number of prior lines of therapy (median, range)	1	(0–4)
Number of metastatic sites (median, range)	1	(0–5)
Performance status at inclusion		
0–1	115	78%
2–3	27	18%
NA	5	3%
Sites of metastasis		
Liver	50	34%
Lung	19	13%
Peritoneum/ovaries	49	33%
Bone	12	8%
LN	74	50%

### Tumour samples and analysis

Of the 147 patients who were consented, complete analysis (CNV and mutations) could be performed for only 81 patients (55%), while mutational analysis alone and CNV analysis alone could be performed for 30 (20%) and 3 (2%) patients, respectively. No analysis could be performed for 33 patients (22%), in the majority of cases due to provision of an inadequate tumour sample (to small) in 18 cases, while insufficient DNA after extraction and insufficient cellularity were the causes of failure in 11 and 4 cases, respectively. With regards to CNV analysis, the switch from an array CGH based technology to and NGS-based technology for CNV assessment drastically changed the efficiency due to a much smaller required amount of DNA (see material and methods). RNA seq was performed for 27 (18%) patients and microsatellite stability was assessed for 37 (25%) patients.

### Recurrent molecular alterations in gastro-oesophageal carcinoma

**Fig. 1.**
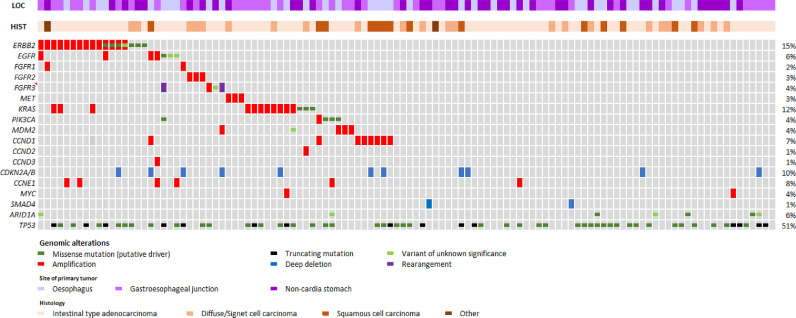
Recurrent molecular alterations in patients with advanced gastroesophageal cancer.

Fig. 1 shows the recurrent molecular alterations identified in 114 patients with at least one molecular analysis (CNV or mutations). As expected and previously reported ERBB2 amplification and mutation were the most common oncogenic events in this cohort (*n* = 17, 15%), followed by KRAS amplification or mutations (*n* = 16, 12%), and CCND1 amplifications (*n* = 8, 7%). Other oncogenic alterations of interest included EGFR amplification and mutations found in 7 patients (5%), MET amplification, found in 3 patients (3%), while FGFR1, 2 and 3 alterations (amplification, mutations and fusions) were found in 9 patients (8%). As previously described, oncogenic alterations in ERBB2, FGFR, MET were found almost exclusively in the adenocarcinoma subtype (regardless of tumour location); On the other hand, EGFR and KRAS alterations as well as CCND1 amplification were found in tumours with both adenocarcinoma and squamous cell carcinoma histology. With regards to tumour suppressors, TP53 was mutated in half of all cases (*n* = 58, 51%), while CDKN2A/B homozygous deletion was the second most common alteration on our panel (*n* = 11, 10%), in both cases irrespective of histology and primary tumour location. Two of 16 (%) patients with adenocarcinoma of the oesophagus were found to have FGFR3 rearrangement by RNA-Seq, while one patient with adenocarcinoma of the gastric antrum had high microsatellite instability (MSI-H).

### Tumour board and treatment recommendation

**Fig. 2.**
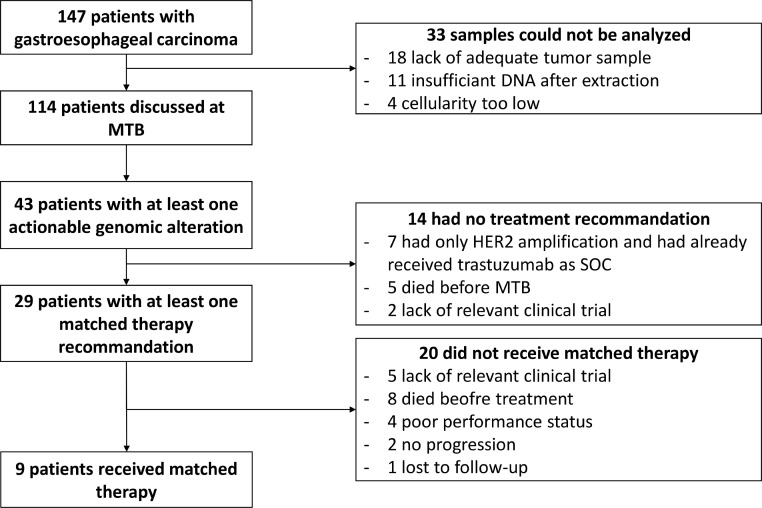
Flow chart of the study.

One hundred and fourteen cases were discussed in MTB (Fig. 2). The median time from inclusion to MTB discussion was 13 weeks (4–52). For thirty patients (26%) the discussion in MTB occurred after the patient had died. Overall, at least one actionable alteration was identified in 43 (38%) patients, including five patients with 2 actionable alteration (in four cases ERBB2 + a co-alteration) and one patient with 3 potentially actionable target. The most frequent alterations were ERBB2 amplification/mutation (*n* = 17/43, 40%), KRAS amplification/mutations (*n* = 11/43, 26%), PIK3CA mutation (*n* = 4, 9%), MDM2 amplification (*n* = 4, 9%) and MET amplification (*n* = 2, 5%)(Fig. 1). All but one patient with ERBB2 amplification identified in this study had concomitant overexpression of HER2 by IHC and had received HER2-targeted therapy as standard of care (fluoropyrimidine and platinum combined with trastuzumab in all cases) prior to molecular tumour board meeting. Molecularly matched therapy was recommended for 29 patients (25% of patients discussed in MTB – *n* = 114), 19% of the whole cohort (*n* = 147)). These 29 patients and the recommended matched therapies are listed in Table 2. Of these 29 patients, 9 went on to receive matched therapy. Their outcome is detailed in Table 3. Amongst these 9 patients, 5 patients had disease control at least equal to that of the previous line of therapy (as shown by a PFS2/PFS2 ratio ≥ 1). Amongst these 5 patients, two were treated with single agent targeted therapy: one patient with an FGFR3 fusion who received futibatinib (TAS-120), an FGFR inhibitor, and one patient with a MET amplification who received crizotinib (a MET inhibitor). Two patients with EGFR amplification who had received 4 prior lines of therapy had disease control lasting 9.7 and 14.3 month with cetuximab and irinotecan-containing chemotherapy. Twenty patients for whom a recommendation was made were not able to initiate molecularly matched therapy for the following reason: deterioration of general condition or rapid disease progression (*n* = 7), no access to relevant therapy (no clinical trial) (*n* = 7), physician decision (*n* = 3), no progression of disease on current line (*n* = 3), one patient received a non-matched experimental therapy and one patient had died (a few days) before the MTB.

**Table 2.** Therapy recommandation according to molecular alterations (*N* = 29). Pat num = patient number (arbitrary). Non-cardia = non-cardia gastric cancer; ADK = intestinal type histology; SIG = signet cell histology, SCC = squamous cell carcinoma. NA = not applicable; FU = follow-up; CR = complete response. ESCAT = ESMO Scale for Clinical Actionability of molecular Targets (Mateo et al. 2018). PFS1: progression-free survival on the last line of therapy prior to the results of molecular analysis.

Pat num	Primary tumour location	Histology	Nb prior lines	PFS1	Actionable alteration	MTB recommandation	ESCAT tier	Treated	Reason not treated	Therapy	PFS on MTA (PFS2)
1	Cardia	ADK	2	2.3	MET amlification	Crizotinib	II	Yes	NA	crizotinib	7.5
2	Non-cardia	SIG	2	2.8	CCND1 amplification	CDK4/6 inhibitor	IV	No	No drug/trial available		
3	Cardia	ADK	5	3.2	ERBB2 amplification	lapatinib	II	Yes	NA	LV5FU2-CDDP+trastuzumab	1.9
4	Cardia	ADK	3	9.4	CCNE1 amplification	pan-CDK inhibitor	IV	No	No drug/trial available		
5	Oesophagus	SCC	4	2.8	PIK3CA hot spot mutation	everolimus	IV	Yes	NA	everolimus	2.8
6	Oesophagus	SCC	3	7.2	EGFR amplification	EGFR inhibitor	IV	Yes	NA	cetuximab+irinotecan	14.8
7	Cardia	SCC	2	11.2	PIK3CA amplification	PI3K/Akt/mTOR inhibitor	IV	No	No drug/trial available		
8	Cardia	ADK	3	3	CDKN2A homozygous deletion	CDK4/6 inhibitor	IV	No	Poor general conditon		
9	Cardia	ADK	2	11.7	CCND1 amplification	CDK4/6 inhibitor	IV	No	No drug/trial available		
10	Oesophagus	SCC	4	1.5	PDGFRB mutation	PAZOPANIB or SORAFENIB or NILOTINIB	IV	No	Poor general conditon		
					PIK3CA hot spot mutation	PI3K/Akt/mTOR inhibitor	IV	No	Poor general conditon		
11	Cardia	ADK	2	2.5	KRAS amplification	sorafenib	IV	No	Poor general conditon		
					KRAS amplification	sorafenib	III				
					MTOR amplification	PI3K/Akt/mTOR inhibitor	IV				
12	Non-cardia	ADK	3	12	BRCA1 mutation	olaparib	III	Yes	NA	olaparib	5.8
13	Cardia	ADK	3	6.4	KRAS amplification	sorafenib	IV	No	Other trial		
14	Non-cardia	ADK	1	4.8	RICTOR amplification	PI3K/Akt/mTOR inhibitor	IV	No			
15	Cardia	SIG	3	2	MET amlification	crizotinib	II	No	Poor general conditon		
16	Cardia	ADK	3	6.5	MDM2 amplification	MDM2 inhibitor	IV	Yes	NA	AMG232	2.8
17	Cardia	ADK	1	54	KRAS amplification	sorafenib	IV	No	CR after last line		
18	Cardia	ADK	2	7	VEGFA amplification	sorafenib	IV	No	lost FU		
					EGFR amplification	EGFR inhibitor	IV				
19	Cardia	ADK	2	29.5	KRAS amplification	sorafenib	IV	No	CR after last line		
20	Oesophagus	ADK	2	8.8	FGFR3 amplification	FGFR inhibitor	II	No	Physician decision		
21	Oesophagus	SIG	2	4.4	KRAS amplification	sorafenib	IV	No	Death		
22	Oesophagus	ADK	4	5.6	MDM2 amplification	MDM2 inhibitor	IV	Yes	NA		
					FGFR3 fusion	FGFR inhibitor	II			TAS-120	7.3
23	Cardia	ADK	2	7.6	NOTCH4 amplification	NOTCH inhibitor	IV	Yes	NA	ABEMACICLIB + LY3039478	3.3
24	Cardia	ADK	6	1.3	KRAS amplification	sorafenib	IV	No	Rapid pogression		
25	Non-cardia	ADK	2	5.8	KRAS amplification	Sorafenib	IV	No	Physician decision		
26	Oesophagus	ADK	2	9	BRCA2 mutation	olaparib	III	No	Poor general conditon		
27	Oesophagus	ADK	4	3	EGFR amplification	EGFR inhibitor	IV	Yes	NA	FOLFIRI+panitumumab	9.5
28	Cardia	ADK	1	20.8	PIK3CA mutation	PI3K/Akt/mTOR inhibitor	IV	No	CR after last line		
29	Cardia	ADK	2	10.3	EGFR amplification	EGFR inhibitor	IV	No	Physician decision

**Table 3.** Outcome of patients who received matched therapy. PFS1 = progression-free survival on the last line of therapy prior to the results of molecular analysis and molecularly matched therapy. PFS2 = progression-free survival on molecularly matched therapy.

Pat num	Primary tumour location	Histology	Nb prior lines	PFS1	Actionable alteration	MTB recommandation	ESCAT tier	Therapy	PFS2	PFS2/PFS1
1	Cardia	ADK	2	2.3	MET amlification	Crizotinib	II	crizotinib	7.5	3.3
3	Cardia	ADK	5	3.2	ERBB2 amplification	lapatinib	II	LV5FU2-CDDP+trastuzumab	1.9	0.6
5	Oesophagus	SCC	4	2.8	PIK3CA hot spot mutation(E542K)	PI3K/Akt/mTOR inhibitor	IV	everolimus	2.8	1.0
6	Oesophagus	SCC	3	7.2	EGFR amplification	EGFR inhibitor	IV	cetuximab+irinotecan	14.8	2.1
12	Non-cardia	ADK	3	12	BRCA1 mutation	olaparib	III	olaparib	5.8	0.5
16	Cardia	ADK	3	6.5	MDM2 amplification	MDM2 inhibitor	IV	AMG232	2.8	0.4
22	Oesophagus	ADK	4	5.6	FGFR3 fusion	FGFR inhibitor	II	TAS-120	7.3	1.3
23	Cardia	ADK	2	7.6	NOTCH4 amplification	NOTCH inhibitor	IV	ABEMACICLIB + LY3039478	3.3	0.4
27	Oesophagus	ADK	4	3	EGFR amplification	EGFR inhibitor	IV	FOLFIRI+panitumumab	9.5	3.2

### Progression-free and overall survival

**Fig. 3.**
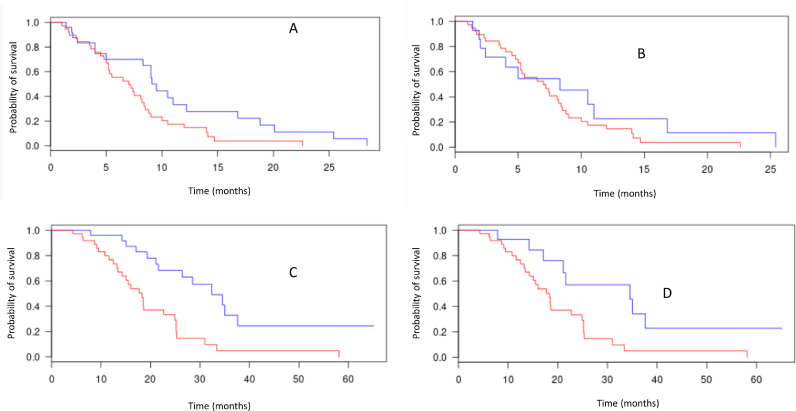
Survival anlyses Panel A: PFS on first line chemotherapy, patients with stage IV at diagnosis with at least one actionable alteration (blue line, *N* = 26) vs no actionable alteration (red line, *N* = 38) (Log rank *p*=.029). Panel B: PFS on first line chemotherapy, patients with stage IV at diagnosis with at least one actionable alteration, excluding 11 patients with ERBB2 amplification (blue line, *N* = 15) vs no actionable alteration (red line, *N* = 38) stage IV patients (Log rank *p*=.302). Panel C: OS from diagnosis according to the presence of an actionable alteration (including ERBB2 amplification, blue line, *N* = 26) vs no actionable alteration (red line, *N* = 38)(*p*=.0003). Panel D: OS from diagnosis according to the presence of an actionable alteration (excluding 11 patient with ERBB2 amplification, blue line, *N* = 15) vs no actionable alteration (blue line, *N* = 38)(*p*=.010);.

In an effort to understand the impact of actionable alterations on response to therapy and survival we analysed the outcome of patients who had stage IV tumours at diagnosis for whom mutation and/or copy number analysis was available (*n* = 64). Their overall survival (OS) since diagnosis was 18.6 months, and there was no difference in OS between squamous cell histology and adenocarcinomas (median 18.6 vs 18.4, respectively, *p* =.62). Progression-free survival on first line therapy was longer for patients whose tumour had at least one actionable alterations vs those who did not (*p* =.029, Fig. 3A), but this difference was no longer significant when patients with HER2+ tumors were excluded (Fig. 3B). Interestingly, OS since diagnosis was significantly longer for patients with actionable alterations (Fig. 3C), even when excluding patients with ERBB2 amplification (Fig. 3D).

### HER2-positive tumours

Co-alterations have been previously described to be associated with intrinsic resistance to trastuzumab in patients with HER2+ gastro-oesophageal cancers [5,17]. Thus we analysed the impact of co-occuring alterations in this subgroup of patient in our cohort. Twenty-one patients had tumours classified as HER2 3+ based on immunohistochemistry. Complete CNV analysis was available for 15 of these patients, while NGS was available for 17, and 14 of these patients had both analyses available. Overall, 14 patients had documented ERBB2 amplification, including one that was considered negative on IHC, while 8 patients had ERBB2 mutations, including 5 with both ERBB2 amplification and mutation (4 activating mutations and 1 variant of unknown significance). Co-alteration of other oncogenic drivers, mainly MET, EGFR and KRAS amplifications were found in 6 patients (Fig. 1). The progression-free survival on chemotherapy (FOLFOX or 5FU+CDDP) + trastuzumab of these patients was not statistically significantly different form that of patients without co-alterations of other oncogenic driver (*p*=.94 – Suppl. Fig. S1).

## Discussion

Gastric and oesophageal cancer are highly heterogeneous with various histological phenotypes and molecular diversity. Inter-patient tumour heterogeneity is an obstacle to identifying optimized targeted therapies in GC, which may in fact vary between molecularly defined subgroups. Indeed, stratification of patients based on tumour genomic alterations may allow the delineation of subgroup-specific therapies, as is already the case for patients with HER2 overexpression [15]. We report here our experience with prospective NGS using an intermediate-size cancer gene panel to guide therapy and identify predictive biomarkers of drug response in patients with gastroesophageal cancer. As previously reported, multiplex sequencing of tumour samples from patients with gastroesophageal cancer is feasible and does identify potentially actionable targets, in most cases amplification of known oncogenes such as ERBB2, EGFR, FGFR1–3, KRAS and MET, in a significant proportion of patients. Interestingly, although some alterations such as ERBB2 and MET amplifications are only seen in adenocarcinomas (both intestinal and signet-cell carcinoma, and in both gastric and oesophageal tumors), EGFR and CCND1 amplifications and PIK3CA alterations were found in patients with both adenocarcinoma and squamous cell histologies. This is important as most of the molecular characterisation in gastroesophageal cancer so far have been done on adenocarcinoma subtypes and identification of patients subgroups with molecularly actionable alterations may help expand the limited treatment options for patients with squamous-cell carcinoma of the oesophagus. As an example, a patient with EGFR amplified SCC of the oesophagus had prolonged tumour control with cetuximab and irinotecan in the present study (Table 3). In this study, the clinical utility of these information was limited by the use of molecular screening occurring too late in patients’ clinical history. As a result, many patients died of disease progression or had poor performance status before the molecular results were available and discussed in molecular tumour board. This can be improved by the earlier use in patient care, of molecular screening tools. Given the limited number of lines available for patients with advanced gastroesophageal cancer and the rapidly progressing course of these tumours, we advocate for the use of molecular screening when the diagnosis of advanced stage is made. Many patients had insufficient archival tumour material to allow adequate analysis (Only 55% had complete CNV and mutational analysis in our series), in most cases diagnostic biopsies were too small to yield enough DNA for analysis. This suggest that changes in clinical practice will be required for the successful use of precision oncology in gastroesophageal cancer. In addition to earlier use of sequencing, the amount of tumour material sampled during endoscopic and percutaneous will need to increase to allow DNA and RNA analyses in addition to conventional diagnostic pathology. Successful early use of molecular screening in gastric cancer was recently reported by investigators from the Samsung Medical center in the VIKTORY trial which was recently reported [8]. The optimal tools for molecular screening is still debated and several molecular screening platforms have received FDA and/or EMA approval. Most of these are comprehensive panels of more than 400 genes, which allow the simultaneous detection of multiple molecular features, including tumour mutational burden (TMB), microsatellite instability (MSI) and oncogenic fusions. Although the optimal use of TMB still requires refinement, the identification of patients with tumours harbouring rare fusions and MSI-tumours is necessary given the response to specific inhibitors and immune checkpoint inhibitors, respectively [7,12,18,21]. Furthermore, given the poor prognosis of gastroesophageal cancer in general, molecular analyses should be implemented at the earliest possible, and possibly at diagnosis, which would allow the use of molecularly guided therapy up-front, including in the preoperative setting. Once again, this will require changes in clinical practice, to increase the amount of tumour sampled during diagnostic procedures to allow molecular subtyping in addition to standard diagnostic pathology. While this may prove difficult up-front, patients with insufficient tumour material may be advised to undergo a new biopsy for additional molecular analyses. Issues around the cost and reimbursement of molecular screening also remains an issue in many countries and ultimately leads to significant treatment inequities. Many have criticized the utility of molecular screening and most trials have so far shown only modest improvement in outcome [13], and this has, in some countries, been used to justify lack or inadequate reimbursement. Another limitation to the utility of molecular screening is the availability of matched therapy. Many compounds are either exclusively investigational or not available “off label” due to cost issues and lack of reimbursement in this indication. This issue has already been highlighted in previous reports of precision medicine in oncology, where, on average, 10% or less of enroled patients do eventually receive matched therapy [6,13]. In addition, despite the relatively small size of our panel, several patients had more than one potentially actionable alteration, which raises the question of combinations or sequential use of targeted therapies. Several reports of successful use of targeted therapy combinations in patients with multiple actionable alterations have been made, mostly in the form of case series [2,16] which is encouraging. Also, as previously reported several patients had tumours with multiple putative oncogenic driver, including patients with ERBB2 amplified tumours that had co-amplification of other oncogenes [5,17]. However, in our series, the duration of response to first line trastuzumab and chemotherapy did not differ between patients with ERBB2 as their sole identified oncogenic driver compared to those with co-alterations, possibly owing to the relatively small number of patients in our cohort. Interestingly, in this study, patients with actionable alterations had longer overall survival than patients who did not. Although our sample size is limited, this effect seemed to be driven by the survival of patients with ERBB2-amplified tumours who all received trastuzumab-based therapy (as expected), but also by the overall survival of patients with other alterations who received matched therapy. This is important as it suggests that molecular screening and the identification of an actionable alteration may alter the course of the disease. As previously reported [11], patients that had EGFR amplification seem to derive significant benefit from the addition of EGFR-blockers to conventional chemotherapy as shown by a PFS2/PFS1 ration of 2 or more, with the limitation of small numbers in our series. This suggests that this subgroup of patient may benefit from a targeted approach as suggested by others [25]. Although various EGFR-targeted therapies have been assessed in patients with advanced gastroesophageal cancer, these data collectively suggest that their development should be reconsidered in a molecularly selected patient subgroup. Similarly, patients with FGFR2 amplification may benefit from the addition of bemarituzumab to chemotherapy as recently reported in a randomized phase 2 study by Wainberg et al. at the 2021 Gastrointestinal Cancer Symposium [24]. For other alterations such as KRAS amplification, PIK3CA mutations or CCND1 amplification, the optimal targeting modality hasn't been established yet, and the safety profile of currently available agents (such as MEK inhibitors, PI3K inhibitors or CDK4/6 inhibitors) will make combination with standard of care chemotherapy challenging from the safety stand point. Still, the current routine molecular screening for HER2 expression using IHC may be extended to EGFR, MET and FGFR and may help incorporating additional therapies in first line regimens, though the confirmation will likely require large scale international efforts as these molecular subgroups are quite rare. In addition, there is permanent refinement in how some alterations can be best targeted. For example, ERBB2-mutant NSCLC were shown to preferentially internalize the HER2 receptor antibody–drug conjugate complex, and this has led to new studies in ERBB2-mutant solid tumors (which include gastric and oesophageal cancers, as shown here)(NCT04639219). Other oncogenic drivers which are currently considered as non-targetable may become actionable in the future. For example, in addition to specific inhibitors of KRAS G12C (which are predominant in NSCLC) inhibitors targeting other frequent substitution of KRAS are getting closer to entering clinical trials (for example MRTX1133 for KRAS G12D, a KRAS mutation frequent in gastrointestinal cancer). Thus, proper tumour sampling and availability of molecular screening tool will be key for the routine management of cancer patients in the near future.

Finally, anti-PD1/PD-L1, have recently shown activity in both squamous and adenocarcinoma subtypes of oesophageal cancer and in gastric cancer, and in most studies a correlation between PD-L1 expression and activity was shown. However, as all the analyses in this cohort pre-date the widespread availability of anti-PD1/PDL1 for these indication, PDL1 expression was not assessed as part of this study nor in routine. Thus we were not able to establish correlation between somatic alterations and PD-L1 expression. In non-small-cell lung cancer, most oncogene addicted subtypes of adenocarcinomas have been shown to be less responsive to immunotherapy [14], but whether this can be translated in gastric or oesophageal cancer remains to be shown.

## Conclusion

In conclusion, molecular screening for actionable alterations should be implemented early in patients with advanced gastroesophageal cancers and this will require better tumour sampling to allow both pathological diagnosis and molecular analyses on the same sample. Access to matched therapy currently remains a significant bottleneck, but the number of approved targeted agents is constantly and rapidly increasing since the early 2000 [10].

## CRediT authorship contribution statement

**Philippe A. Cassier:** Conceptualization, Writing – original draft, Data curation, Formal analysis. **Clémentine Peyramaure:** Data curation, Formal analysis. **Valery Attignon:** Writing – original draft. **Lauriane Eberst:** Writing – original draft. **Camille Pacaud:** Data curation, Formal analysis. **Sandrine Boyault:** Writing – original draft. **Françoise Desseigne:** Writing – original draft. **Mathieu Sarabi:** Writing – original draft. **Pierre Guibert:** Writing – original draft. **Pauline Rochefort:** Writing – original draft. **Nathalie Marques:** Writing – original draft. **Michel Rivoire:** Writing – original draft. **Aurélien Dupré:** Writing – original draft. **Patrice Peyrat:** Writing – original draft. **Catherine Terret:** Writing – original draft. **Isabelle Ray-Coquard:** Writing – original draft. **Clélia Coutzac:** . **David Pérol:** Conceptualization. **Jean-Yves Blay:** Conceptualization. **Olivier Trédan:** Conceptualization. **Christelle de la Fouchardière:** Writing – original draft.

## Declaration of Competing Interest

The authors declare the following financial interests/personal relationships which may be considered as potential competing interests:

## Appendix Supplementary materials

Image, application 1

## References

[1] Arnold M., Ferlay J., van Berge Henegouwen M.I., Soerjomataram I. (2020). Global burden of oesophageal and gastric cancer by histology and subsite in 2018. Gut.

[2] Aubanel M., Swalduz A., Avrillon V., Doublet L., Mastroianni B., Neidhardt-Bérard E.M., Pérol M. (2020). Combining EGFR and MET inhibition with crizotinib in EGFR-mutated lung adenocarcinoma harboring MET amplification: a brief report. Clin. Lung Cancer.

[3] Cancer Genome Atlas Research Network (2014). Comprehensive molecular characterization of gastric adenocarcinoma. Nature.

[4] Cancer Genome Atlas Research Network (2017). Integrated genomic characterization of oesophageal carcinoma. Nature.

[5] Janjigian Y.Y., Sanchez-Vega F., Jonsson P., Chatila W.K., Hechtman J.F., Ku G.Y., Riches J.C., Tuvy Y., Kundra R., Bouvier N., Vakiani E., Gao J., Heins Z.J., Gross B.E., Kelsen D.P., Zhang L., Strong V.E., Schattner M., Gerdes H., Coit D.G., Bains M., Stadler Z.K., Rusch V.W., Jones D.R., Molena D., Shia J., Robson M.E., Capanu M., Middha S., Zehir A., Hyman D.M., Scaltriti M., Ladanyi M., Rosen N., Ilson D.H., Berger M.F., Tang L., Taylor B.S., Solit D.B., Schultz N. (2018). Genetic predictors of response to systemic therapy in esophagogastric cancer. Cancer Discov..

[6] Le Tourneau C., Delord J.P., Gonçalves A., Gavoille C., Dubot C., Isambert N., Campone M., Trédan O., Massiani M.A., Mauborgne C., Armanet S., Servant N., Bièche I., Bernard V., Gentien D., Jezequel P., Attignon V., Boyault S., Vincent-Salomon A., Servois V., Sablin M.P., Kamal M., Paoletti X., SHIVA investigators (2015). Molecularly targeted therapy based on tumour molecular profiling versus conventional therapy for advanced cancer (SHIVA): a multicentre, open-label, proof-of-concept, randomised, controlled phase 2 trial. Lancet Oncol..

[7] Le D.T., Durham J.N., Smith K.N., Wang H., Bartlett B.R., Aulakh L.K., Lu S., Kemberling H., Wilt C., Luber B.S., Wong F., Azad N.S., Rucki A.A., Laheru D., Donehower R., Zaheer A., Fisher G.A., Crocenzi T.S., Lee J.J., Greten T.F., Duffy A.G., Ciombor K.K., Eyring A.D., Lam B.H., Joe A., Kang S.P., Holdhoff M., Danilova L., Cope L., Meyer C., Zhou S., Goldberg R.M., Armstrong D.K., Bever K.M., Fader A.N., Taube J., Housseau F., Spetzler D., Xiao N., Pardoll D.M., Papadopoulos N., Kinzler K.W., Eshleman J.R., Vogelstein B., Anders R.A., Diaz L.A. (2017). Mismatch repair deficiency predicts response of solid tumors to PD-1 blockade. Science.

[8] Lee J., Kim S.T., Kim K., Lee H., Kozarewa I., Mortimer P.G.S., Odegaard J.I., Harrington E.A., Lee J., Lee T., Oh S.Y., Kang J.H., Kim J.H., Kim Y., Ji J.H., Kim Y.S., Lee K.E., Kim J., Sohn T.S., An J.Y., Choi M.G., Lee J.H., Bae J.M., Kim S., Kim J.J., Min Y.W., Min B.H., Kim N.K.D., Luke S., Kim Y.H., Hong J.Y., Park S.H., Park J.O., Park Y.S., Lim H.Y., Talasaz A., Hollingsworth S.J., Kim K.M., Kang W.K. (2019). Tumor genomic profiling guides patients with metastatic gastric cancer to targeted treatment: the VIKTORY umbrella trial. Cancer Discov..

[9] Liu Y., Sethi N.S., Hinoue T., Schneider B.G., Cherniack A.D., Sanchez-Vega F., Seoane J.A., Farshidfar F., Bowlby R., Islam M., Kim J., Chatila W., Akbani R., Kanchi R.S., Rabkin C.S., Willis J.E., Wang K.K., McCall S.J., Mishra L., Ojesina A.I., Bullman S., Pedamallu C.S., Lazar A.J., Sakai R., Thorsson V., Bass A.J., Laird P.W., Cancer Genome Atlas Research Network (2018). Comparative molecular analysis of gastrointestinal adenocarcinomas. Cancer Cell.

[10] Lu D., Lu T., Stroh M., Graham R.A., Agarwal P., Musib L., Li C.C., Lum B.L., Joshi A. (2016). A survey of new oncology drug approvals in the USA from 2010 to 2015: a focus on optimal dose and related postmarketing activities. Cancer Chemother Pharmacol..

[11] Maron S.B., Alpert L., Kwak H.A., Lomnicki S., Chase L., Xu D., O'Day E., Nagy R.J., Lanman R.B., Cecchi F., Hembrough T., Schrock A., Hart J., Xiao S.Y., Setia N., Catenacci D.V.T. (2018). Targeted therapies for targeted populations: anti-EGFR treatment for EGFR-amplified gastroesophageal adenocarcinoma. Cancer Discov..

[12] Marabelle A., Le D.T., Ascierto P.A., Di Giacomo A.M., De Jesus-Acosta A., Delord J.P., Geva R., Gottfried M., Penel N., Hansen A.R., Piha-Paul S.A., Doi T., Gao B., Chung H.C., Lopez-Martin J., Bang Y.J., Frommer R.S., Shah M., Ghori R., Joe A.K., Pruitt S.K., Diaz L.A. (2020). Efficacy of pembrolizumab in patients with noncolorectal high microsatellite instability/mismatch repair-deficient cancer: results from the phase II KEYNOTE-158 study. J. Clin. Oncol..

[13] Massard C., Michiels S., Ferté C., Le Deley M.C., Lacroix L., Hollebecque A., Verlingue L., Ileana E., Rosellini S., Ammari S., Ngo-Camus M., Bahleda R., Gazzah A., Varga A., Postel-Vinay S., Loriot Y., Even C., Breuskin I., Auger N., Job B., De Baere T., Deschamps F., Vielh P., Scoazec J.Y., Lazar V., Richon C., Ribrag V., Deutsch E., Angevin E., Vassal G., Eggermont A., André F., Soria J.C (2017). High-throughput genomics and clinical outcome in hard-to-treat advanced cancers: results of the MOSCATO 01 trial. Cancer Discov..

[14] McLean L., Leal J.L., Solomon B.J., John T. (2021). Immunotherapy in oncogene addicted non-small cell lung cancer. Transl. Lung Cancer Res..

[15] Oh D.Y., Bang Y.J (2020). HER2-targeted therapies - a role beyond breast cancer. Nat. Rev. Clin. Oncol..

[16] Piotrowska Z., Isozaki H., Lennerz J.K., Gainor J.F., Lennes I.T., Zhu V.W., Marcoux N., Banwait M.K., Digumarthy S.R., Su W., Yoda S., Riley A.K., Nangia V., Lin J.J., Nagy R.J., Lanman R.B., Dias-Santagata D., Mino-Kenudson M., Iafrate A.J., Heist R.S., Shaw A.T., Evans E.K., Clifford C., Ou S.I., Wolf B., Hata A.N., Sequist L.V. (2018). Landscape of acquired resistance to osimertinib in EGFR-mutant NSCLC and clinical validation of combined EGFR and RET inhibition with osimertinib and BLU-667 for acquired RET fusion. Cancer Discov..

[17] Sanchez-Vega F., Hechtman J.F., Castel P., Ku G.Y., Tuvy Y., Won H., Fong C.J., Bouvier N., Nanjangud G.J., Soong J., Vakiani E., Schattner M., Kelsen D.P., Lefkowitz R.A., Brown K., Lacouture M.E., Capanu M., Mattar M., Qeriqi B., Cecchi F., Tian Y., Hembrough T., Nagy R.J., Lanman R.B., Larson S.M., Pandit-Taskar N., Schöder H., Iacobuzio-Donahue C.A., Ilson D.H., Weber W.A., Berger M.F., de Stanchina E., Taylor B.S., Lewis J.S., Solit D.B., Carrasquillo J.A., Scaltriti M., Schultz N., Janjigian Y.Y. (2019). EGFR and MET amplifications determine response to HER2 inhibition in ERBB2-amplified esophagogastric cancer. Cancer Discov..

[18] Schram A.M., Chang M.T., Jonsson P., Drilon A. (2017). Fusions in solid tumours: diagnostic strategies, targeted therapy, and acquired resistance. Nat. Rev. Clin. Oncol..

[19] Sicklick J.K., Kato S., Okamura R., Schwaederle M., Hahn M.E., Williams C.B., De P., Krie A., Piccioni D.E., Miller V.A., Ross J.S., Benson A., Webster J., Stephens P.J., Lee J.J., Fanta P.T., Lippman S.M., Leyland-Jones B., Kurzrock R. (2019). Molecular profiling of cancer patients enables personalized combination therapy: the I-PREDICT study. Nat. Med..

[20] Stransky N., Cerami E., Schalm S., Kim J.L., Lengauer C. (2014). The landscape of kinase fusions in cancer. Nat. Commun..

[21] Subbiah V., Yang D., Velcheti V., Drilon A., Meric-Bernstam F. (2020). State-of-the-Art strategies for targeting RET-dependent cancers. J Clin Oncol.

[22] Trédan O., Wang Q., Pissaloux D., Cassier P., de la Fouchardière A., Fayette J., Desseigne F., Ray-Coquard I., de la Fouchardière C., Frappaz D., Heudel P.E., Bonneville-Levard A., Fléchon A., Sarabi M., Guibert P., Bachelot T., Pérol M., You B., Bonnin N., Collard O., Leyronnas C., Attignon V., Baudet C., Sohier E., Villemin J.P., Viari A., Boyault S., Lantuejoul S., Paindavoine S., Treillleux I., Rodriguez C., Agrapart V., Corset V., Garin G., Chabaud S., Pérol D., JY; Blay (2019). ProfiLER investigators. Molecular screening program to select molecular-based recommended therapies for metastatic cancer patients: analysis from the ProfiLER trial. Ann Oncol..

[23] Varnier R., Le Saux O., Chabaud S., Garin G., Sohier E., Wang Q., Paindavoine S., Pérol D., Baudet C., Attignon V., Pissaloux D., Heudel P., You B., Leyronnas C., Collard O., Trédan O., Bonnin N., Long J., Jacquin J.P., Cassier P.A., Derbel O., Freyer G., Viari A., Blay J.Y., Ray-Coquard I (2019). Actionable molecular alterations in advanced gynaecologic malignancies: updated results from the ProfiLER programme. Eur. J. Cancer.

[24] Wainberg Z.A., Enzinger P.C., Kang Y.K., Yamaguchi K., Qin S., Lee K.W., Oh S.C., Li J., Turk H.M., Teixeira A.C., Cardellino G.G., Guardeño R., Mitra S., Yang Y., Collins H., Catenacci D.V.T (2021). Randomized double-blind placebo-controlled phase 2 study of bemarituzumab combined with modified FOLFOX6 (mFOLFOX6) in first-line (1L) treatment of advanced gastric/gastroesophageal junction adenocarcinoma (FIGHT). J Clin Oncol.

[25] Strickler J. (2018). *EGFR* Amplification as a target in gastroesophageal adenocarcinoma: Do anti-EGFR therapies deserve a second chance?. Cancer Discov..

