# Supplementary material for: Precision medicine for patients with gastro-oesophageal cancer: A subset analysis of the ProfiLER program
Source: Transl Oncol. 2021 Nov 15;15(1):101266. doi: 10.1016/j.tranon.2021.101266 (PMC8605190; doi:10.1016/j.tranon.2021.101266)
Supplement: Supplementary file 1 [file mmc1.docx]

**Supplementary data and figures**

**Suppl. data 1 : FusionPlex RNA CTL (Archerdx) :**

Mutations (targeted exons) : AKT1 (3) , ALK (21 à 25), AXL (5, 11, 15, 17), BRAF (11, 15) , CTNNB1 (3), DDR2 (17) , EGFR (18, 19, 20, 21), ERBB2 (20), FGFR2 (8*), FGFR3 (7, 9*, 10*), GNAS (8, 9), HRAS (2, 3, 4), IDH1 (4), IDH2 (4), KIT (11, 13, 17), KRAS (2, 3, 4), MAP2K1 (2, 3) , MET (14 à 20), NRAS (2, 3, 4), POLE (9 à 14), PIK3CA (9, 20), RET (11, 13, 14, 15, 16), ROS1 (38)

Fusions : ALK, AXL, BRAF, CCND1, FGFR1, FGFR2, FGFR3, MET, NRG1, NTRK1, NTRK2, NTRK3, PPARG, RAF1, RET, ROS1

Overexpression : ALK, CCND1, EGFR, ERBB2, FGFR1, FGFR2, FGFR3, KIT, MET, NTRK1, NTRK2, NTRK3, RET, ROS1


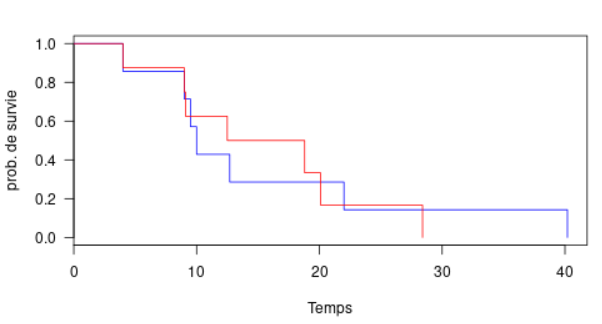


**Supplementary Figure S1**: Progression-free survival on first line trastuzumab-containing chemotherapy of patients with HER2 3+ tumors, with (red line) or without co-alterations (blue line) (p=.94).
